# Astragalin, a Flavonoid from *Morus alba* (Mulberry) Increases Endogenous Estrogen and Progesterone by Inhibiting Ovarian Granulosa Cell Apoptosis in an Aged Rat Model of Menopause

**DOI:** 10.3390/molecules21050675

**Published:** 2016-05-21

**Authors:** Min Wei, Gail B. Mahady, Daniel Liu, Zhi S. Zheng, Ye Lu

**Affiliations:** 1 Institute of Botany, Jiangsu Province and Chinese Academy of Sciences, Nanjing 210041, China; wm96403@sina.com (M.W.); zzheng@sina.com (Z.S.Z.); lvyenji@yahoo.com(Y.L.); 2 Department of Pharmacy Practice, College of Pharmacy, University of Illinois at Chicago, Chicago, IL 60612, USA; mahady@uic.edu; 3 Beijing Clinical Services Center, No. 103 Chaoyang North Road, Beijing 100123, China;

**Keywords:** astragalin, apoptosis, Bax, Bcl-2, herbal medicine, mechanism, menopause

## Abstract

*Background*: To determine the mechanism by which the flavonoid glycoside astragalin (AST) reduces ovarian failure in an aged rat model of menopause. *Methods*: The *in vivo* effect of AST on granulosa cell (GC) apoptosis in aged female rats was determined using flow cytometry. *In vitro*, the effects of AST on cultured GCs were investigated using the MTT proliferation assay and western blot assays. *Results*: Aged rats had significantly higher GC apoptosis as compared with young female rats. Treatment of aged rats with AST (all three doses; *p* < 0.01) or Progynova (*p* < 0.01) significantly reduced GC apoptosis as compared with the aged controls. The proportions of total apoptotic GCs was 25.70%, 86.65%, 47.04%, 27.02%, 42.09% and 56.42% in the normal, aged, 17β-estradiol (E_2_), high dose AST, medium dose AST, and low dose AST-treated groups, respectively. Significant increases of serum E_2_ and P_4_ levels, as well as altered levels of serum follicle stimulating hormone (FSH) and luteinizing hormone (LH) levels. In cultured rat GCs, AST stimulated GC proliferation, E_2_ and progesterone (P_4_) secretion, reduced apoptosis, reduced the level of the pro-apoptotic protein Bcl-2 (*p* < 0.01), but had no effect on BAX. *Conclusions*: AST enhanced ovarian function in aged female rats by increasing E_2_ and P_4_ levels, and reducing ovarian GC apoptosis via a mechanism involving Bcl-2. These data demonstrate a new pharmacological activity for AST, as well as a novel mechanism of action, and further suggest that AST may be a new therapeutic agent for the management of menopausal symptoms.

## 1. Introduction

Reproduction senescence is a naturally occurring phenomenon that occurs in all female mammals, regardless of species [1]. For women, the transition to the menopause is characterized by a progressive decline in fertility associated with the loss of ovarian follicles, a reduction in oocyte quality, age-related defects in the uterus, as well as changes in neuroendocrine function [2,3]. Also associated with the transition are gradual reductions in hormone profiles; irregular cycles; declining fertility with age; disturbances in thermogenesis; and age-related gains in body weight and fat distribution [2,3]. Investigations into the cellular and molecular mechanisms of ovarian failure that precipitate menopause include assessments of follicle aging and granulosa cell apoptosis [2,3]. Follicular ageing is characterized by the impairment of specific functions of oocytes and granulosa cells (GCs), as well as general cellular dysfunction, including reduced mitrochondrial activity, energetic failure, and changes in gene and protein expression [2,3]. Since GCs are involved in the conversion of androgens to estrogens, as well as progesterone synthesis, a reduction in their number and function leads to reduced levels of endogenous E_2_ and P_4_ and hence, menopausal symptoms [4,5]. The death of GCs by apoptosis triggers follicular atresia, and it appears that both E_2_ and IGF-I suppress apoptosis in GCs during follicular atresia [4,5]. Granulosa cells are the main source of estradiol, and IGF-I increases the secretion of estradiol from GCs [5]. Thus, therapies that reduce GC apoptosis and increase endogenous E_2_ and P_4_ levels represent novel therapeutic options for menopausal women.

In the US, menopause occurs in women between the ages of 40 and 60 years, with an average age of ~52 years [1]. Approximately 55%–75% of women entering menopause exhibit both vasomotor and neurological symptoms, including hot flushes, sleep disorders, irritability, depression and anxiety, which when severe, can cause a significant decline in their health and quality of life [1,6,7]. In severe cases, these symptoms lead many women to seek medical attention. Treatment with hormone therapy (HT) has been the gold standard for menopause, however HT has been associated with an increased risk of adverse events, including a transitory increased risk of heart disease, and increased risk of endometrial or breast cancer [8,9,10,11,12]. On the basis of the risks and findings from the Women’s Health Initiative, the U.S. Preventive Services Task Force has published a recommendation against the routine use of HT for the prevention of chronic conditions in postmenopausal women [12]. As a consequence, many women worldwide are looking for safe and effective alternative therapies to manage their menopausal symptoms.

Herbal medicines, including traditional Chinese medicines (TCM), are used in most countries worldwide for the management of menopausal symptoms [13,14,15,16,17,18,19]. In a pilot observational clinical trial, we have demonstrated that treatment of menopausal Chinese women with a TCM formula named Menoprogen (MPG) that contains five medicinal herbs, including *Morus alba* L*.* (Moraceae), significantly reduced the Kupperman index [15]. In addition, treatment with this formula increased the endogenous levels of E_2_ and P_4_. Furthermore, in an aged rat model of menopause, treatment with the same TCM formula, reduced ovarian granulosa cell (GC) apoptosis via a mechanism involving IGF-I, and thereby enhanced the levels of endogenous of E_2_ and P_4_ in these animals [14,19,20,21].

**Figure 1:**
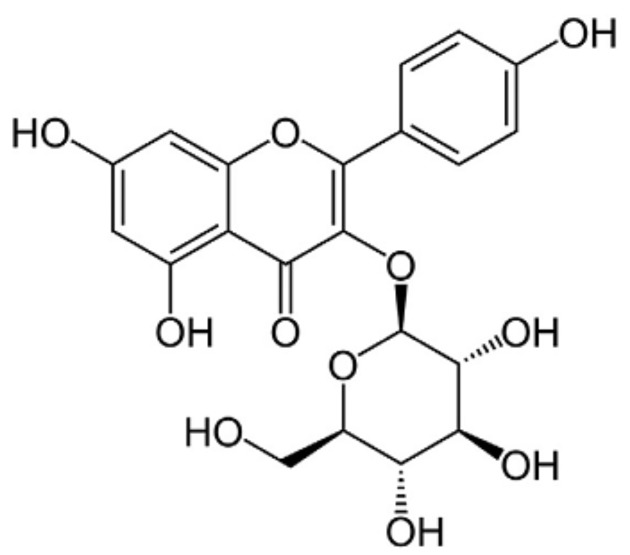
The chemical structure of Astragalin (AST), a flavonoid glycoside present in the traditional Chinese herbal medicine *Morus alba* (mulberry).

In an attempt to identify the active chemical constituents of the MPG formula that may be responsible for these effects on granulosa cells, we have investigated some of the primary chemical compounds present in *M. alba* fruits. The main flavonoid glycosides include quercetin, astragalin, isoquercetin and rutin [16,17,18]. In this work, we demonstrate that purified astragalin (kaempferol-3-*O*-glucoside; Figure 1), a flavonoid glycoside that isolated from *M. alba* (white mulberry fruit) one of the herbal constituents of the TCM formula for menopause reduces ovarian GC apoptosis *in vitro* and *in vivo* and reverses ovarian failure in an aged female rat model of menopause. This compound appears to be one of the active chemical constituents in the MPG formula, and responsible, at least in part, for its pharmacological effects on menopausal symptoms.

## 2. Results

### 2.1. AST Does Not Alter Uterine, Ovarian and Pituitary Indices in Aged Female Rats

**Figure 2:**
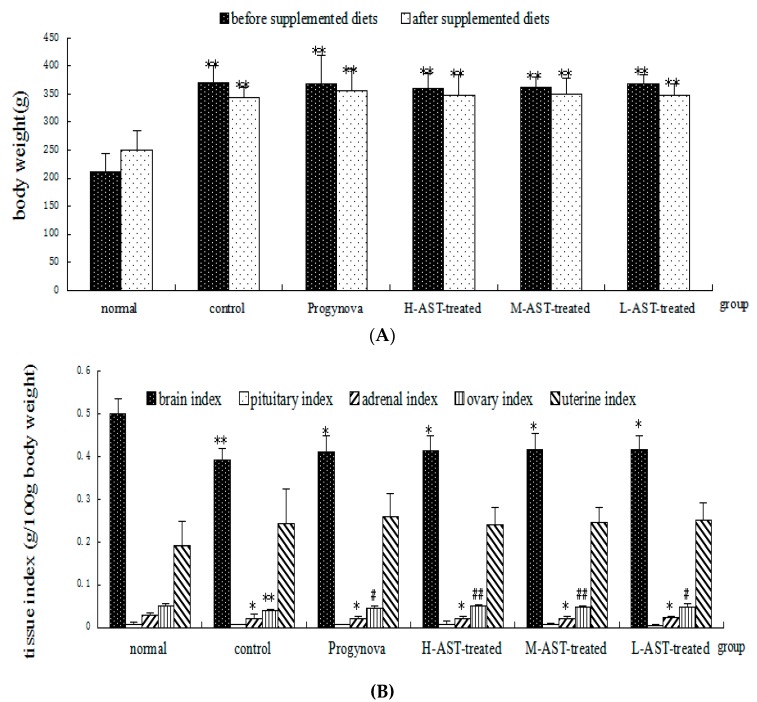
Effect of AST on the (**A**) body weight and (**B**) tissue indices of aged senile rats (*n* = 8 per arm). Normal group, non-aged (treated with vehicle); control group, aged (treated with vehicle); Progynova group, treated with Progynova 0.18 mg/kg; H-AST-treated group, M-AST-treated group, and L-AST-treated group, treated with AST 70, 35 and 7 μmol/kg, i.g., respectively. Results are presented as mean ± SEM. * *p* < 0.05, ** *p* < 0.01, control group, Progynova group, H-AST-treated group, M-AST-treated group, and L-AST-treated group* versus* normal group; ^#^ *p* < 0.05, ^##^ *p* < 0.01, Progynova group, H-AST-treated group, M-AST-treated group, and L-AST-treated group *versus* control group.

The initial body weights of all five aged groups were similar and did not statistically vary. The body weights increased continuously in all groups. At the end of the study, the rats in the Progynova group and the three AST-treated groups showed no differences in body weight, as compared with the aged control group (Figure 2A; *p* > 0.05). As expected, the brain, ovarian and adrenal indices (determined as g/100 g body weight) were significantly reduced in the aged 14-month control group compared with the normal 4-month old rats (*p* < 0.01, *p* < 0.01 and *p* < 0.05, respectively), showing that ageing results in atrophy of the brain, ovary and adrenal. However, pituitary and uterine indices of the aged control group were not significantly different from normal group (*p* > 0.05). Treatment of the aged rats with Progynova, high dose AST, medium dose AST, or low dose AST reduced the ovary index loss as compared with the aged control group (*p* < 0.05,* p* < 0.01, *p* < 0.01, and *p* < 0.05, respectively) but still resulted in a lower ovary index than that of normal group (*p* > 0.05; Figure 2B).

### 2.2. AST Reduces Apoptosis of Ovarian GCs in Aged Female Rats

**Figure 3:**
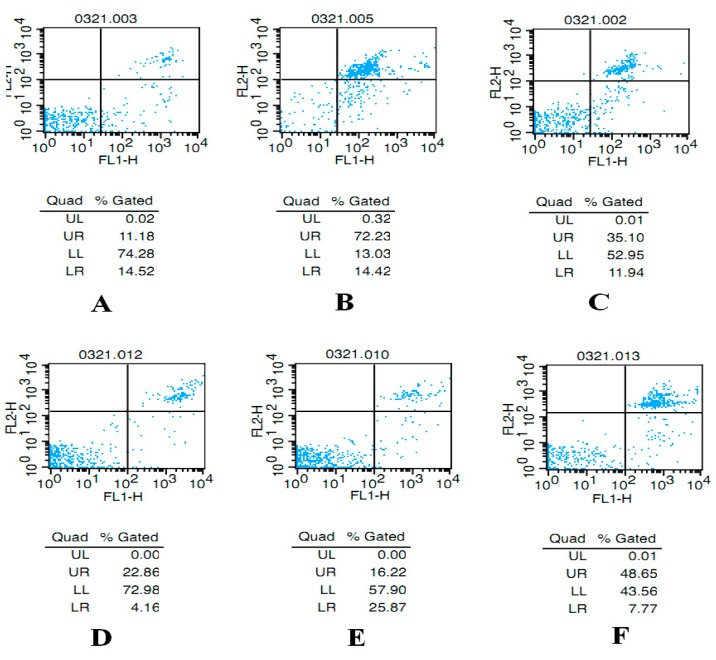
Reduction of ovarian granulosa cell apoptosis in aged female rats after treatment with increasing concentrations of AST. Apoptosis rate was determined by flow cytometry after staining with Annexin-V-FITC and propidium iodide. (**A**) Normal group, non-aged (treated with vehicle); (**B**) control group, aged (treated with vehicle); (**C**) Progynova group, treated with Progynova 0.18 mg/kg; (**D**) H-AST-treated group, treated with AST 70 μmol/kg; (**E**) M-AST-treated group, treated with AST 35 μmol/kg; (**F**) L-AST-treated group, treated with AST 7 μmol/kg.

As shown in Figure 3A–F, UL represent the proportions of dead cells; UR represent the proportions of late apoptotic cells; LL represent the proportions of living cells; LR represent the proportions of early apoptotic cells. After administration, the proportions of total apoptotic cells (late and early apoptotic cells) for the ovarian GCs were 25.70%, 86.65%, 47.04%, 27.02%, 42.09% and 56.42% in normal group (Figure 3A), control group (Figure 3B), Progynova group(Figure 3C), H-AST-treated group (Figure 3D), M-AST-treated group (Figure 3E), L-AST-treated group (Figure 3F), respectively. 

**Figure 4:**
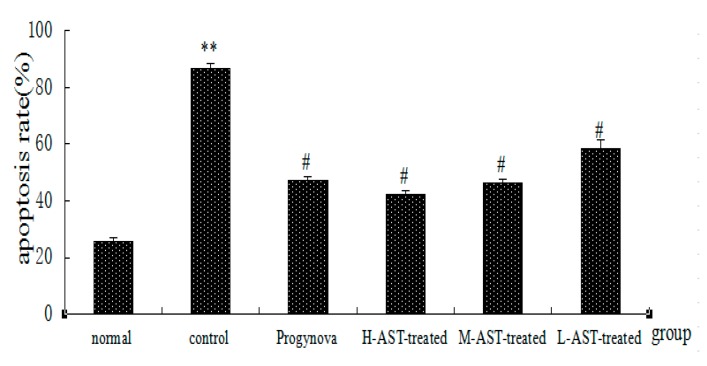
Effect of AST on the apoptosis rate of ovarian granulosa cells in aged female rats (*n* = 3 per arm). Apoptosis rate % = the number of apoptosis cells / (the number of apoptosis cells + the number of normal cells) × 100%. Normal group, non-aged (treated with vehicle); control group, aged (treated with vehicle); Progynova group, treated with Progynova 0.18 mg/kg; H-AST-treated group, M-AST-treated group, and L-AST-treated group, treated with AST 70, 35 and 7 μM/kg, i.g., respectively. Results are presented as mean ± SEM. ** *p* < 0.01, control group, Progynova group, H-AST-treated group, M-AST-treated group, and L-AST-treated group versus normal group; ^#^ *p* < 0.05, Progynova group, H-AST-treated group, M-AST-treated group, and L-AST-treated group versus control group.

As shown in Figure 4, the control group had the highest apoptosis rate (*p* < 0.01), which was coincident with the apoptosis of ovarian GCs. It was also evident that three doses of AST (*p* < 0.01) and Progynova (*p* < 0.01) administration reduced the apoptosis of ovarian GCs in aged female rats as compared with those of control group. Moreover, the data suggest that low doses of AST were superior to Progynova in reducing apoptosis.

### 2.3. AST Increases Hormone Production in Aged Female Rats 

**Figure 5:**
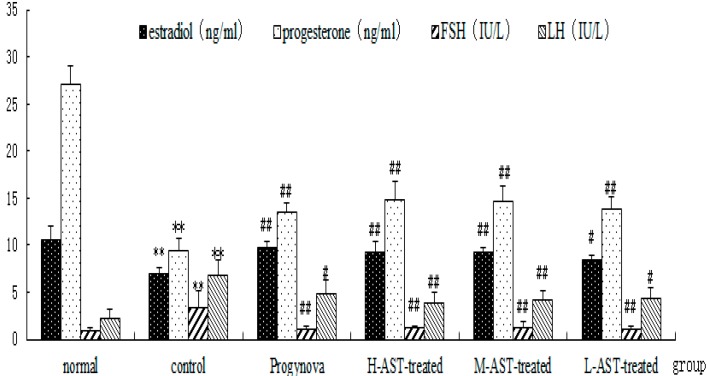
Effect of AST on hormone levels in the sera of aged senile rats (*n* = 8 per arm). Normal group, non-aged (treated with vehicle); control group, aged (treated with vehicle); Progynova group, treated with Progynova 0.18 mg/kg; H-AST-treated group, M-AST-treated group, and L-AST-treated group, treated with AST 70, 35 and 7 μM/kg, respectively. Results are presented as mean ± SEM ** *p* < 0.01, control group, Progynova group, H-AST-treated group, M-AST-treated group, and L-AST-treated group versus normal group; ^#^ *p* < 0.05, ^##^ *p* < 0.01, Progynova group, H-AST-treated group, M-AST-treated group, and L-AST-treated group versus control group.

The initial hormone levels of the five groups (except the normal 4-month old rat group) were similar (Figure 5). The effects of AST on the mean plasma levels of E_2_, P_4_, FSH and LH are shown in Figure 5. The aged control group had the lowest levels of E_2_ and P_4_, and the highest levels of FSH and LH (*p* < 0.01), which were coincident with the gradual failure of ovulation, as well as hypo-secretion or deficiency of sex hormones in aged control rats. Treatment with Progynova or AST alone significantly increased the levels of serum E_2_ (*p* < 0.01) and P_4_ (*p* < 0.01), and reduced the levels of FSH (*p* < 0.05) and LH (*p* < 0.05) in the aged control rats compared with normal group. Moreover, the effects of AST on the levels of P_4_ and LH were slightly better than those of Progynova, however this was not statistically significant.

### 2.4. AST Increases GC Proliferation in Cultured GCs

**Figure 6:**
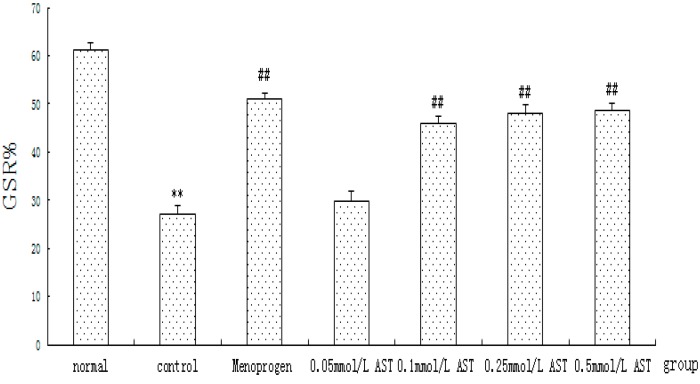
Effect of AST on the cellular viability of cultured ovarian GC measured by MTT assay (*n* = 30). Cells were incubated in absence or presence of AST at different concentrations. Growth stimulation ratios (GSR) were calculated using the following equation: GSR% = (A_sample_− A_normal_) / A_normal_ × 100, where A is the average absorbance of all wells. The experiment was repeated five times, six-well/each time. Results are presented as mean ± SEM. ** *p* < 0.01, control group versus normal group; ^##^ *p* < 0.01, all treatment groups versus control group.

The effects of AST on cellular proliferation of cultured GCs were quantified using the MTT assay (Figure 6). The results showed that cellular proliferation of ovarian GCs was significantly reduced to 27.2% in the CdCl_2_ treated control group as compared with 61.3% in the normal group after incubation with CdCl_2_, indicating that the cells were experiencing apoptosis (*p* < 0.01). AST induced a typical concentration-dependent increase in GC cellular proliferation. The cells treated with 0.05 mM AST 24 h only showed a slight increase of GC cellular viability (*p >* 0.05) but a significant increase was observed in cells treated with 0.1 mM to 0.5 mM AST (*p* < 0.01) and Menoprogen (*p* < 0.01). No significant differences were observed among the drug groups.

### 2.5. AST Increases GC Hormone Production and Secretion in Cultured GCs

**Figure 7:**
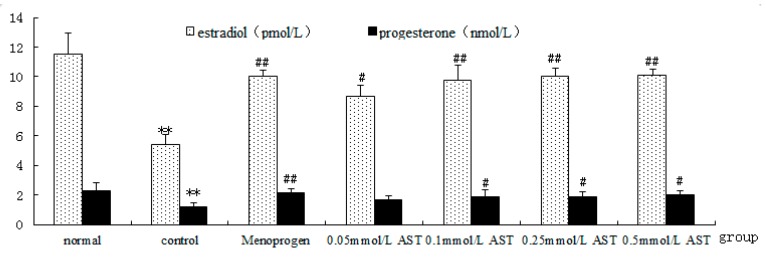
Effect of AST on hormone secretory activity of cultured GC measured by RIA assay (*n* = 10). Normal describes GCs from normal 4-month old female rats; control represents GCs from the naturally aged 14-month old rats. Isolated GCs were incubated in absence or presence of increasing concentrations of AST or Menoprogen. The experiment was repeated five times, two-well/each time. Results are presented as mean ± SEM. ** *p* < 0.01, control group versus normal group; ^#^ *p* < 0.05, ^##^ *p* < 0.01, all treatment groups versus control group.

Effects of AST on hormonal secretory activity of cultured GC were quantified by RIA assay (Figure 7). Results showed that GCs from aged rats had a reduced hormone production and secretion (*p* < 0.01). However, AST or Menoprogen treatment of the GCs increased both E_2_ and P_4_ production in a concentration-dependent manner (Figure 7) and AST (0.1 mM to 0.5 mM) and Menoprogen significantly increased E_2_ (*p* < 0.01) production. Moreover, the enhancement of P_4_ (*p* < 0.01) by Menoprogen was superior to that of AST(*p* < 0.05).

### 2.6. AST Reduced Apoptosis in Cultured GCs

**Figure 8:**
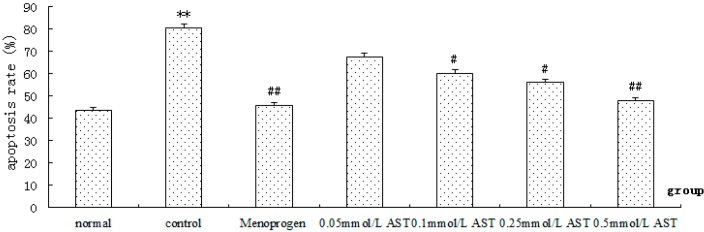
Effect of AST on apoptosis of ovarian GC measured by flow cytometry (*n* = 10 per arm). Apoptosis rate % = the number of apoptosis cells / (the number of apoptosis cells + the number of normal cells) × 100%. Cells were incubated in absence or presence of AST at different concentrations. The experiment was repeated five times, in duplicate. Results are presented as mean ± SEM. ** *p* < 0.01, control group versus normal (4-month old rats) group; ^#^ *p* < 0.05, ^##^ *p* < 0.01, all treatment groups versus control (NAM 14-month old rats) group.

Apoptotic index was determined by flow cytometry. After a 24 h treatment with increasing concentrations of AST, a concentration-dependent decrease of apoptotic cells was observed (Figure 8). The results suggested that AST (0.1 mM to 0.5 mM) and Menoprogen significantly decreases CdCl_2_-induced apoptosis in ovarian GC apoptosis (*p* < 0.05), so these three doses were used for the remaining studies.

### 2.7. AST Alters the Ratio of Bcl-2 and Bax Proteins in Cultured GCs

**Figure 9:**
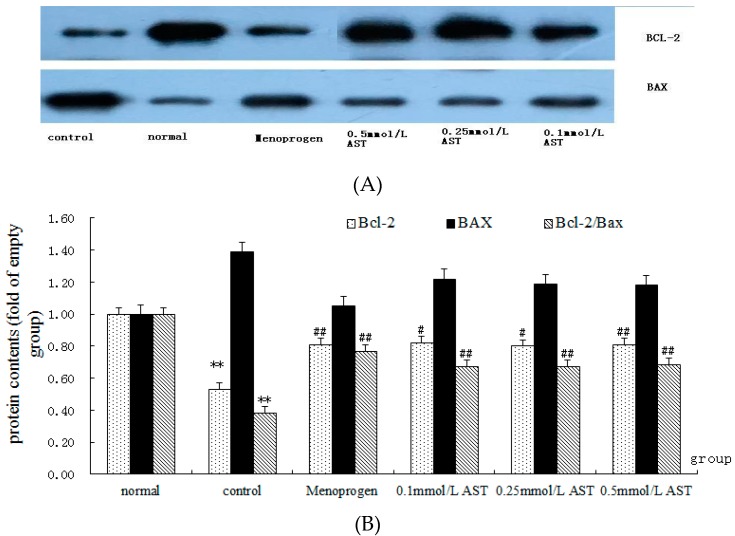
(**A**) Western blot analysis of AST on apoptosis-related proteins of ovarian GCs. (**B**) Effect of AST on apoptosis-related proteins of ovarian GCs as determined by Western Blot analysis (*n* = 12). Cells were incubated in absence or presence of AST at different concentrations. The experiment was repeated three times using four replicates each time. Results are presented as mean ± SEM. ** *p* < 0.01, control group versus normal group;^#^ *p* < 0.05, ^##^ *p* < 0.01, all treatment groups versus control group.

The number of the copies of Bcl-2 protein and the ratio of Bcl-2 mRNA/Bax proteins were significantly decreased in cultured GCs after incubated with CdCl_2_ (*p* < 0.01, Figure 9). The results suggest that CdCl_2_ increases ovarian GC apoptosis by reducing the synthesis of Bcl-2 and by increasing the production of Bax. AST (0.1 mM to 0.5 mM) and MPG significantly (*p* < 0.05) increased the expression of Bcl-2 protein and the ratio of Bcl-2 mRNA/Bax mRNA (*p* < 0.01). No significant difference in the expression of Bax was observed when the GCs were treated with AST at any concentration (0.1 mM, 0.25 mM, 0.5 mM) and MPG, compared to control group (all *p* > 0.05), indicating AST produced no significant effect on Bax.

## 3. Discussion

Since the publication of the Women’s Health Initiative (WHI) in 2002 [6,7], there has been an increased interest in complementary and alternative medicine (CAM) treatments for menopause, including herbal therapies. However, reviews of the clinical literature indicate that the evidence supporting the use of herbal medicines for the treatment of menopause is inconclusive and often contradictory [22]. Furthermore, the mechanism(s) by which most herbal therapies act to reduce menopausal symptoms remains elusive, although several hypotheses have been suggested [23]. Understanding the mechanisms of action of herbal medicines, as well as the active chemical constituents responsible for these effects will lead to better quality herbal products and more adequately designed clinical trials, as well as a better understanding of the patient populations that should be recruited for such studies. These data would also be invaluable in the assessment of safety for these products. 

Since 2008 we have been investigating a TCM herbal formula Menoprogen (MPG) [14,15,16,17,18,19], which has been shown to reduce menopausal symptoms and the Kupperman index in Chinese women [15]. This formula contains five medicinal and food plants, and has been used in China for centuries to treat menopausal symptoms [14,15,16,17,18,19]. In our previous work, we have shown that the mechanism of MPG is very novel in that it does not act as a phytoestrogen, but works indirectly by reducing GC apoptosis via a mechanism involving IGF-1. By reducing GC apoptosis, MPG enhances GC viability and the production of endogenous E_2_ and P_4_, which in turn reduces menopausal symptoms [14]. However, the active chemical constituents of the formula had not been previously identified. One of the five plants in MPG is *M. alba*, which has also been used in TCM for thousands of years for the treatment of a wide range of women’s reproductive disorders including breast cancer, dysmenorrhea, and menopause [15]. We have tested some of the primary chemical constituents from *M. alba* for their effects on GC apoptosis * in vitro* and *in vivo*. One of these compounds, AST, also known as kaempferol-3-*O*-glucoside is one of the primary flavonoid glycosides isolated from mulberry leaves and fruits, and is reported to be present in the plant in concentrations up to 9.5 mg/g [24,25,26,27]. Previous studies of AST have shown that this compound has anti-inflammatory, anti-autophagy, anti-apoptosis, antioxidant, and anti-atopic effects [24,25,26,27,28,29,30,31]. However, AST has not previously investigated for its effect on ovarian failure or GC apoptosis. In this study, we demonstrate that AST inhibited GC apoptosis both * in vitro* and *in vivo*, thereby maintaining the viability of GCs and enhancing their production of endogenous E_2_ and P_4_, and reducing ovarian failure in an aged female rat model of menopause. Thus, its mechanism of action is similar to that of MPG, suggesting that AST is at least one of the active chemical constituents of the MPG formula. In support of the anti-apoptotic effects of AST, Cho* et al.*, [28,29] have reported that AST reduced apoptosis in BEAS-2B epithelial airway cells by modulating oxidative stress-responsive MAPK signaling [28]. In our study, the mechanism by which AST reduced GC apoptosis involved a significant increase in the levels of the anti-apoptotic protein, Bcl-2, which then led to a significant alteration of the Bcl-2/Bax ratio in the cells. Bax, a pro-apoptotic protein that is expressed in both GCs and oocytes, and plays a central role in ovarian cell death, and the ovarian lifespan of GCs can be extended by reducing Bax or altering the Bcl-2/Bax ratio [32,33]. Our results support those of Qu* et al.*, [27] who demonstrated that AST reduced apoptosis in heart tissues of rats after myocardial ischemia and reperfusion. This group also used western blot analysis and showed that the reduction in apoptosis in heart tissues by AST was due to a significant reduction in Bax, and an increase in Bcl-2 protein levels [27]. In our work, we found AST did not decrease Bax protein expression, and only increased Bcl-2 levels. This may be explained by the fact that we were using different cell types than Qu *et al.*, [27], and the methods used were slightly different.

## 4. Materials and Methods

### 4.1. Chemicals

**Figure 10:**
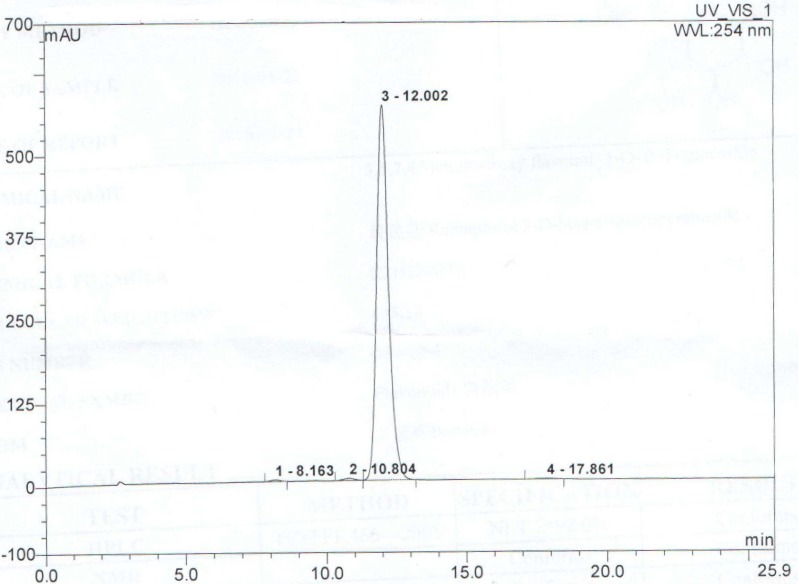
HPLC chromatogram of purified AST and its retention time (min).

Purified AST (purity > 99.9%) was obtained from the National Institute for the Control of Pharmaceutical and Biological Products (Beijing, China) and was then dissolved in phosphate-buffered saline (PBS) to make a solution of 0, 0.05, 0.1, 0.25, 0.5 mM for the * in vitro* testing, and also dissolved into diluted in normal saline (1% sodium carboxymethylcellulose-CMC-Na) for the *in vivo* testing. Menoprogen herbal formula was exactly as described previously [14]. HPLC of AST (Figure 10) was performed using previously described methods [34]. The separation was performed on a Hypersil BDS C18 column (4.6 × 150 mm, 5 mm) with a C18 guard column. The elution was performed using a gradient solvent system with 1% glacial acetic acid (solvent A) and methanol (solvent B) as the mobile phases. The ratios were as follows: 80:20 (A/B) for 3 min, 80:20 to 65:35 (A/B) in 11 min, held for 14 min, 65:35 to 0:100 (A/B) in 25 min and held for 5 min. The flow rate was 1.0 mL/min. The PDA detector was monitored at 334 nm and the injection volume for all samples and standards was 10 mL. 

### 4.2. Effects of AST on GC Apoptosis In Vivo 

#### 4.2.1. Naturally Aged Rat Model of Menopause

A total of 48 Sprague-Dawley female rats (SPF grade; Sino-British Sippr/BK Lab, Shanghai, China) were included in the study [14,19]. Forty of these rats were 14 months old (250–300 g), while the other eight were 4 months old (180–220 g). The animals were maintained under regulated environmental conditions (temperature, 22 ± 2 °C; humidity, 45%–55%; 12 h light/dark cycle) and provided with a standard diet and water *ad libitum*. The animal protocols were approved in accordance with the UK Animals (Scientific Procedures) Act of 1986 and its associated guidelines, number A11062. The 14-month-old SD female rats were observed for four continuous estrous cycles using a smear with vaginal exfoliated cells followed after staining with hematoxylin that were obtained between 9:00 a.m. and 10:00 a.m.. The ageing female model was established using a vaginal cytological technique involving an extended estrogenic cycle, continuous estrous, and repeated fake pregnancy in cells.

#### 4.2.2. Experimental Design

The 14-month-old SD female rats as the ageing model were randomly divided into five groups (8 rats/group): vehicle-treated (control group), Progynova-treated (Progynova group, 0.18 mg/kg), low dose AST-treated (L-AST-treated group, 7 μM/kg), medium dose AST-treated (M-AST-treated group, 35 μM/kg) and high dose AST-treated (H-AST-treated group, 70 μM/kg). The reference daily intake for Progynova (17β-estradiol; Sigma-Aldrich, St Louis, MO, USA) is 2 mg/day. Converting the clinically equivalent dose for a 70-kg adult to that for a 200-g rat by body surface area ratio, the dose of Progynova for rats is 0.18 mg/kg/day. Menoprogen (MPG) contains five semi-purified plant extracts, including *M. alba* [14,15,16,17,18,19]. The polyphenol content of *M. alba* is approximately 32 mg/g, of which ~9.5 mg/g is astragalin [35,36]. MPG contains five herbal extracts of which 20% is *M. alba* [14,19]. MPG is administered at a dose of 0.4 g/capsule, with 2 to 4 capsules per day [14,15,16,17,18,19,20]. The flavonoid content of MPG is approximately 20% per capsule, of which ~80 mg is AST. The doses of AST are determined by an *in vivo* pretest study. Converting the effective dose of AST for a 70-kg adult (2.5 mg/kg or 175 mg/day) to that of a 200 g rat using body surface area ratio, the dose is 15.76 mg/kg. We used this as the middle dose. For the high dose we used two times the middle dose and for the low dose we used 10 times below the high dose, giving 30 mg/kg and 3 mg/kg, respectively. So three different doses of AST are 70, 35 and 7 μmol/kg in rats. Four-month-old SD female rats as healthy controls were given vehicle (normal group). All samples were suspended in normal saline (CMC-Na). Rats in the control group and normal group were administered the same volume of normal saline. Rats were all administered the test substances through an oral gavage for 2 weeks. The dosing was adjusted according to rat weight on a weekly basis.

Blood samples that were collected from the inferior vena cava before and after administration were centrifuged at 3000 rpm for 15 min at 4 °C and stored at −20 °C for serum analyses at the time of diestrus. At the end of the treatment period, all of the rats were sacrificed after taking blood, the brain, bilateral ovaries, bilateral uterine, adrenal gland and pituitary glands were removed and weighed to determine the tissue indices. Thereafter, the ovarian tissue was rinsed with PBS at 37 °C and granulosa cells were isolated from medium (2–5 mm diameter) antral follicles by fine-needle aspiration according to the method of Channing and Ledwitz-Rigby [22] for cytometry analysis.

#### 4.2.3. Histological Analysis 

The brain indices, adrenal indices, uterine indices, ovarian indices and pituitary indices were defined respectively as the weight ratio of these tissues to the rat body to which they belonged (g/100 g body weight).

#### 4.2.4. Apoptosis Assay

The ovarian granulosa cells were maintained in DMEM/F12 medium at a concentration of 1 × 10^6^ cells/mL, and were re-suspended in 400 μL of annexin V binding buffer containing fluorescein isothiocyanate (FITC)-conjugated annexin V (Mbchem, Shanghai, China). After 15 min of incubation at 37 °C, 10 μL of propidium iodide (PI, 25 μg/mL) was added. The cell suspension was immediately analyzed using fluorescence activated cell sorter (FACS) Calibur flow cytometer (BD Biosciences, San Jose, CA, USA). The excitation wavelength was set at 488 nm with the emission wavelength set at 513 and 675 nm for detecting FITC-annexin V (FL1 channel) and PI (FL3 channel), respectively. Apoptosis rate was calculated by the equation:

Apoptosis rate % = the number of apoptosis cells / (the number of apoptosis cells  + the number of normal cells) × 100% as described by the manufacturer
(1)

#### 4.2.5. Radioimmunoassay of Hormone Levels

Progesterone (P_4_) was determined using a commercially available Radioimmunoassay (RIA) kit (Beijing North Institute of Biotechnology, Beijing, China) with a sensitivity of 0.25 ng/mL. The mean intra- and inter-assay coefficients of variation (CVs) were 5.5% and 9.2%, respectively. The 17β-estradiol (E_2_) was measured using a commercially available RIA kit (Beijing North Institute of Biotechnology) with the sensitivity of 2.5 pg/mL. The mean intra- and interassay CVs were 4.6% and 11.5%, respectively. Follicle-stimulating hormone (FSH) was estimated in plasma samples using a commercially available RIA kit (MP Biomedicals Co., Ltd., Shanghai, China). The sensitivity of the assay was 0.28 IU/L. The mean intra- and inter-assay CVs were 4.8% and 10.2%, respectively. Luteinizing hormone (LH) was estimated in plasma samples using a commercially available RIA kit (Shanghai Kaibo Biochemical Reagent Co., Ltd., Shanghai, China). The sensitivity of the assay was 0.21 IU/L. The mean intra- and inter-assay CVs were 4.2% and 9.0%, respectively.

### 4.3. Effects of AST on GC Apoptosis In Vitro

#### 4.3.1. Ovarian Granulosa Cell Culture

The SD female rats aged 21–25 days old were treated with pregnant mare serum gonadotropin (66 IU/rat, Ningbo Sansheng Pharmaceutical Co., Ltd., Ningbo, China) [22]. After 48 h, the animals were sacrificed, ovaries excised and transported to the laboratory at 4 °C. Ovaries were washed in sterile saline solution, and the follicular fluid was aspirated from 3 to 5 mm follicles, and granulocytes (GCs) were isolated by centrifugation for 10 min at 200× *g*, followed by washing in sterile DMEM/F12 medium (1:1, *v*/*v*, HyClone, Thermo Fisher Scientific Inc., Beijing, China) and re-suspended in the same medium supplemented with 10% fetal calf serum (HyClone) and 1% antibiotic-antimycotic solution (Sigma-Aldrich) at a final concentration of 10^6^ cells/mL medium. Portions of the cell suspension were dispensed into 12-well culture plates (1.5 mL/well; medium for RIA and cells for flow cytometry), 16-well chamber slides (1.0 mL/well, for western blot assay) or 96-well culture plates (10 μL/well, for MTT assay). The experiments were performed in duplicate and repeated five times. Cells were maintained at 37 °C in an incubator with a water-saturated 5% CO_2_ environment. Cultured ovarian GCs from the control group were prepared with the treatment of 600 µM/L cadmium chloride (CdCl_2_), and the normal (negative control) group was treated with vehicle solvent only. 

4.3.2. 3-(4,5-Dimethylthiazol-2-yl)-2,5-Diphenyltetrazolium Bromide (MTT) Proliferation Assay

GCs were plated in 96-well plates at 5 × 10^4^ cells/mL (200 mL/well) and grown in DMEM/F12 medium supplemented with 10% fetal calf serum, which were randomly allocated to five treatment groups (0.1 g Menoprogen, 0.05 mM AST, 0.1 mM AST, 0.25 mM AST and 0.5 mM AST) and one normal PBS group (6 well/group). After 24 h, the cells were washed with PBS. The cells of the treatment groups were cultured with serum-free medium containing Menoprogen or AST at increasing concentrations and the cells of normal group were cultured with the same volume of PBS. After 20 h of co-culturing, 20 μL MTT solution (5 mg MTT/mL PBS) was then added into the wells, and the incubation continued for additional 4 h. Finally, MTT solution was removed followed by addition of DMSO (150 μL/well). The absorbance was recorded on a xMark™ Microplate Absorbance Spectrophotometer (Bio-Rad, Hercules, CA, USA) at a wavelength of 595 nm with a reference at 655 nm. Growth stimulation ratios (GSR) were calculated using the following equation:

GSR% = (A_sample_ − A_normal_) / A_normal_ × 100 (2)

where A is the average absorbance of all wells.

#### 4.3.3. Radioimmunoassay Measurements of Estradiol and Progesterone in Cultured GCs

GCs were plated in 12-well plates at 1 × 10^6^ cells/mL (1.5 mL/well), which were randomly allocated to five treatment groups and one normal PBS group (2 well/group) and cultured with serum-free medium. After 24 h, the cells in the control groups were cultured with Menoprogen or AST at increasing concentrations and the cells in the normal group were cultured with the same volume of PBS. Having been incubated for additional 24 h, the cultured GCs were assayed for estradiol and progesterone levels. P_4_ and E_2_ were estimated in plasma samples using the method described above. 

#### 4.3.4. Annexin V Apoptosis Assay

Following the experiments above, the remaining cells in 12-well culture plates were harvested after removing the medium and apoptosis was determine using the annexin V assay as described above. 

#### 4.3.5. Western Blot Assays 

Granulosa cells were cultured in 16-well plates at 1 × 10^6^ cells/mL (1.0 mL/well), and were randomly allocated to Menoprogen group, three AST groups and one normal PBS group (4 well/group), and cultured in serum-free medium. After 24 h, the cells in the control groups were cultured with AST at increasing concentrations (0.05–0.5 mmol) and the cells in the normal group were cultured with the same volume of PBS. After additional incubation for 24 h, the cells were washed twice and then processed with 20 μL of NP-40 lysis buffer containing protease inhibitors. A BCA method was used to determine the protein concentration in each well. Ten to 20 μg of whole cell extract from each well was loaded on each corresponding lane of 4% to 10% polyacrylamide gel. The Tricine-SDS-PAGE polyacrylamide gel electrophoresis was carried out with the voltage setting at 60 V for the concentrated gel and 150 V for the separating gel. A wet-transfer system (Mini Trans-Blot Cell, Bio-Rad Laboratories, Inc.) was used to transfer the protein contents from the gel to the membrane (PVDF, 200 mA for 60 min). After incubation with 5% bovine serum albumin (HyClone, dissolved in PBS) for 2 h at room temperature, blocking the non-specific bindings, the membrane was incubated with one of the primary antibodies, rabbit polyclonal antibody to Bcl-2 (1:500, Abcam, local agency of Nanjing Saiyan Bioengineering Institute, Nanjing, China), to GAPDH (1:500) or to Bax (1:500, Santa Cruz Biotechnology, Inc., local agency of Nanjing Saiyan Bioengineering Institute, Nanjing, China) at 4 °C overnight. The membrane was incubated with the secondary antibody (goat-anti-rabbit HRP conjugated antibody, 1:1500, Santa Cruz Biotechnology, Inc.) at 4 °C for 2 h. The ECL chemical luminescence method was employed to detect and analyzed the target protein using the Gel Image Analysis System and ChemiDoc MP Imaging System (Bio-Rad). Changes in protein level were determined using the Bio-Rad’s stain-free technology and presented as fold of the normal group. The experiment was repeated three times.

### 4.4. Statistical Analysis

Results are presented as mean ± standard error of the mean (SEM) of the observations. Between-group statistical differences in body weight were calculated by factorial analysis of variance (ANOVA), followed by Student’s t test. Two-way factorial ANOVA was performed, with time and treatment as independent categorical variables and with effect of treatment as dependent variable. Values were considered significant at *p* < 0.05. Statistical analysis was performed using one-way ANOVA followed by post hoc analysis using Tukey’s honestly significant difference test (Figure 1, Figure 2 and Figure 3). Statistical analysis was performed using IBM SPSS Statistics for Windows 2015. Student’s *t* test was also used to evaluate the differences between various experimental and control groups. The level of significance was set at a *p* value of 0.05.

## 5. Conclusions

Overall, this work suggests that AST is one of the active constituents of *M. alba* and the MPG formula, as it reduces GC apoptosis by increasing in the levels Bcl-2, leading to a significant alteration of the Bcl-2/Bax ratio in the cells. Since traditional Chinese herbal medicines are usually complex mixtures of multiple herbs, it is likely that there are multiple compounds in the MPG formula that have efficacy, and some may be synergistic. The limitation of this study is that we are measuring the effects of only one compound in this work. In addition, enhancement of endogenous estrogens by AST may contribute to the development of estrogen-responsive cancers. However, interestingly published reports suggest the opposite, that in fact AST, a compound present in many plant species, has chemopreventative and anti-cancer effects [26,37,38,39]. Our own work has shown that MPG itself does not impact estrogen-responsive cancers [18]. Obviously prior to any human study, further toxicological testing would be needed to confirm these results. However, this work also demonstrates a number of important general points that are pertinent to the investigation of treatments for menopause. First, the ovaries of aged female rats undergo many of the changes that are also observed in human ovaries during menopause, making the aged rat model an excellent model for this type of study. Second, follicular atresia and GC apoptosis may be suppressed by treatment with estradiol or AST, which has positive implications for fertility research. Finally, in terms of menopause, the naturally occurring flavonoid AST has a very novel mechanism of action in that it is not directly estrogenic, but works indirectly by suppressing GC apoptosis in aged female rats, thereby reducing ovarian failure and increasing the natural production of E_2_ and P_4_. An increase in endogenous hormone levels would reduce menopausal symptoms, thus indicating that AST alone may be potential treatment for menopause.
